# The Bacillus Agnostic

**Published:** 1911-02

**Authors:** 


### The Bacillus Agnostic.

In from his rural dominion,
Fresh from his rustic location,
Came the bacillus agnostic,
Rank disbeliever in microbes.
Talked he right bold to the doctors—
Those who believe in the ptomaines
Thus spake Reub sore on such folly:
"Long years ago in the country
Had we a place called a schoolhouse;
Always without ventilation,
Ever without any safeguard
'Gainst the onslaught of disease.
Never an oven for pencils,
Never a book fumigator,
Never a bit of precaution
Other than such as the beasts have.
(Some asafetida surely
Eke, some small baglets of sulphur.)
Drank all we brats from one tin cup,
Aye, from one bucket we guzzled;
Some even poured back their leavings!
Sometimes a slate that was borrowed,
Bearing nine kinds of dried moisture,
Had to be cleaned before using—
Used we our tongues for the cleansing.
Yet there was none of us ailing;
Ne'er had we heard of the microbe."

—*Indianapolis Medical Journal.*

